# Recent Advances in Humoral Immunity Dysregulation in Severe Fever With Thrombocytopenia Syndrome Virus (SFTSV) Infection: A Systematic Review

**DOI:** 10.1002/hsr2.71328

**Published:** 2025-10-15

**Authors:** Hongyan Hou, Xu Yuan, Rujia Chen, Zhigang Xiong, Shiji Wu, Feng Wang

**Affiliations:** ^1^ Department of Laboratory Medicine, Tongji Hospital, Tongji Medical College Huazhong University of Science and Technology Wuhan China

**Keywords:** B cell, humoral immunity, immune dysregulation, severe fever with thrombocytopenia syndrome virus (SFTSV)

## Abstract

**Background and Aims:**

Severe fever with thrombocytopenia syndrome virus (SFTSV) infection causes profound dysregulation of humoral immunity, leading to immunopathology and impaired viral clearance. However, the mechanisms underlying B cell dysfunction and inadequate antibody responses remain poorly defined. This review aims to summarize current evidence on B cell‐mediated immune dysregulation in SFTS and explore potential therapeutic implications.

**Methods:**

We conducted a comprehensive review of recent literature on humoral immunity in SFTS, including original research articles, autopsy reports, in vitro and single‐cell RNA‐sequencing studies. We focused on B cell subset alterations, antibody production kinetics, T follicular helper (Tfh) dysfunction, germinal center disruption, and viral immune evasion mechanisms.

**Results:**

SFTSV preferentially targets B cells, especially plasmablasts, which become hyperactivated and serve as potential viral reservoirs. Fatal cases show excessive plasmablast expansion but fail to mount virus‐specific IgG responses. Disrupted germinal center formation, impaired Tfh function, and a shift toward extrafollicular antibody responses contribute to low‐affinity antibody production and increased autoantibody generation. Cytokine storms further suppress B and Tfh cell function. Neutralizing antibody levels correlate with survival, and delayed IgG or absent anti‐Gn responses are associated with poor prognosis.

**Conclusion:**

SFTSV requires humoral immunity by inducing pathological plasmablast proliferation, impairing Tfh‐B cell interactions, and suppressing protective antibody responses. Understanding these mechanisms provides insight into SFTS pathogenesis and highlights potential therapeutic targets such as germinal center restoration, plasmablast modulation, and precision immunotherapy. Future strategies should focus on vaccine design targeting conserved viral epitopes and interventions that preserve long‐lived B cell immunity.

## 1 Introduction

Severe fever with thrombocytopenia syndrome (SFTS) is an emerging infectious disease caused by Dabie bandavirus, also known as severe fever with thrombocytopenia syndrome virus (SFTSV) [1]. SFTSV infection typically causes high fever, thrombocytopenia, leukopenia and gastrointestinal symptoms. In severe cases, it may progress to shock, disseminated intravascular coagulation (DIC) multi‐organ failure and death [2, 3, 4, 5]. Since its discovery in China in 2010, SFTSV has primarily affected East Asia countries, with significant outbreaks reported in China, South Korea, and Japan [6, 7, 8]. SFTSV is mainly transmitted through tick bites, with *Haemaphysalis longicornis* ticks acting as the principal vector [9, 10]. Human‐to‐human transmission has also been documented, typically through contact with infected blood, bodily fluids or respiratory droplets [11, 12, 13]. In 2017, the World Health Organization (WHO) listed SFTSV among the top 10 priority pathogens due to the absence of effective vaccines and antiviral therapies.

The host immune response to SFTSV involves both innate and adaptive immune mechanisms. Innate immunity is primarily mediated by macrophages and dendritic cells, which serve as the first line of defense [14]. In contrast, the adaptive immune response, particularly humoral immunity mediated by B cells, plays a pivotal role in virus clearance and long‐term protection. During SFTSV infection, B cell recognize and internalize antigens via B cell receptors (BCRs) and present them to CD4^+^ T cells through major histocompatibility complex (MHC) Class II molecules. Activated B cells either differentiate into plasma cells that secret immunoglobulin M (IgM) antibodies or migrate to lymphoid follicles, where they interact with follicular dendritic cells and T helper cells to initiate germinal center (GC) formation for somatic hypermutation and affinity maturation [15, 16]. However, accumulating evidence suggests that SFTSV disrupts normal B cell activation, resulting in excessive immune responses and impaired antibody production, thereby exacerbating disease severity [17, 18]. Understanding the dysregulation of humoral immunity during SFTSV infection is essential for identifying immunopathogenic mechanisms. This review aims to synthesize current findings on how SFTSV rewires humoral immunity responses, creating a paradox of B cell hyperactivation coupled with functional antibody deficiency, and discusses emerging therapeutic strategies to restore immune balance.

## 2 B Cell Subpopulations Changes

Recent studies have demonstrated a significant elevation in total B cell counts in patients with SFTS compared to healthy controls, with B cell levels positively correlating with disease severity [18, 19, 20]. Detailed profiling of B cell subpopulations has revealed distinct immunophenotypic patterns between survivors and non‐survivors. In fatal cases, there is a marked expansion of double‐negative (DN) B cells (CD27^−^IgD^−^) and plasmablast/plasma cells (CD38^+^CD27^+^IgD^−^), whereas memory B cells (CD27^+^IgD^+^) are significantly reduced. Although naïve B cells (CD27^−^IgD^+^) are generally decreased across all patients, their levels are comparatively higher in survivors [17, 21]. In peripheral blood sample from SFTS patients, atypical lymphocytes have been identified as activated B cells undergoing differentiation into plasmablasts [22]. A large and transient population of plasmablasts is commonly observed in the peripheral blood of severe cases [23]. Histopathological analyses further confirm that plasmablasts infiltrate secondary lymphoid organs in patients who succumbed to the disease [24]. In vitro studies have demonstrated that peripheral blood mononuclear cells (PBMCs) from SFTS patients can propagate these atypical lymphocytes in response to cytokines, such as tumor necrosis factor‐alpha (TNF‐α) and interleukin‐6 (IL‐6) secreted by SFTSV‐infected B cells, rather than direct stimulation by viral particles [25]. Collectively, these findings indicate substantial disruption in B cell maturation and functional differentiation, contributing to humoral immune dysregulation in fatal SFTS cases.

## 3 Excessive Plasmablasts Proliferation During SFTSV Infection

B cells are considered major targets of SFTSV infection [26]. Suzuki et al. [24] demonstrated that B cells differentiating into plasmablasts and macrophages within secondary lymphoid organs are frenquently infected by SFTSV, as evidenced by autopsy samples from fatal cases [24]. Flow cytometry analyses revealed a markedly higher infection rate in plasmablasts following ex vivo infection of PBMCs [25]. Furthermore, single‐cell RNA sequencing (scRNA‐seq) of PBMCs from SFTS patients identified plasma cells as the predoninant cell type harboring SFTSV RNA. Notably, although only 23 plasma cells contained viral transcripts, this suggests a selected infection pattern. SFTSV infection induces Type I interferon (IFN‐I) responses and upregulated IFN‐stimulated genes (ISGs) to restrict viral replication However, viral non‐structural proteins (e.g., NSs) suppress IFN‐I signaling [27, 28], leading to diminished antiviral responses in infected plasmablasts compared to uninfected B cells [29]. Interestingly, Li. et al. [30] reported that exacessive IFN‐I signaling is associated with hyperinflammation and poor prognosis in SFTS patients, identifying intermediate monocytes (CD14^++^CD16^+)^ and IFN‐I‐inducible plasmablasts as major viral targets [30]. The simutaneous expansion of plasmablasts and absence of virus‐specific immunoglobulin G (IgG) in deceased patients suggestss a profound collapse of B cell mediated immunity [17].

These virus‐associated plasmablasts may serve as reservoirs facilitating viral persistence and immune evasion. To date, only a few cellular receptors for SFTSV entry receptors for SFTSV have been identified. Dendritic cell specific intercellular adhesion molecule‐3‐grabbing nonintegrin (DC‐SIGN), a C‐type lectin receptor, facilitates endocytic entry for SFTSV, Rift Valley fever virus (RVFV), and Uukuniemi virus (UUKV) [31, 32]. SFTSV glycoproteins Gn and Gc mediate viral entry via interaction with host cell factors. DC‐SIGN is essential for Gn/Gc‐dependent infection of monocyte‐derived dendritic cells, yet many permissive cell lines, including B cells, lack endogenous DC‐SIGN expression, suggesting the existence of alternative receptors [33]. A genome‐wide CRISPR‐Cas‐9 screening study identified C‐C chemokine receptor 2 (CCR2) as a key host factor mediating SFTSV entry. CCR2 binds the SFTSV Gn protein via its N‐terminal extracellular domain, with binding dependent on tyrosine sulfation at position Y26 [34]. CCR2 is highly expressed in monocytes and functions as a receptor for chemokine ligand CCL2. Monocytes, are primary SFTSV targets, contribute substantially to inflammatory responses and viral dissemination [30, 35]. PBMCs from elderly individuals and patients with diabetes mellitus exhibit elevated surface CCR2 expression, enhancing viral susceptibility. Notably, plasmablasts and the H929 cell line also express high levels of CCR2, rendering them especially vunerable to SFTSV infection [36]. These findings suggest that therapeutic strategies targeting CCR2 could potentially mitigate viral replication and inflammation in SFTSV infected patients.

Overproliferation of plasmablasts in SFTS is often associated with elevated lambda to kappa light chain ratio, mirroring immunophenotypes observed in multiple myeloma and correlating with disease progression [37, 38]. Although monoclonal lambda‐type plasma cells may transiently appear in the bone narrow, this reactive plasmacytosis should not be misinterpreted as lambda light chain multiple myeloma. Several viruses, including Hantavirus, Epstein‐Barr virus (EBV) and Dengue virus, are known to induce polyclonal B cell activation through pattern recognition receptors and cytokine cascades [39]. Similar findings have shown that Dengue virus can directly activate memory B cells and drive differentiation into antibody‐secreting cells, suggesting that B cells cam act as alternative target cells [40, 41]. However, the precise mechanisms driving the selective expansion of monoclonal lambda‐type plasma cells in SFTSV infection remain unclear.

## 4 Imbalance Between GC and Extrafollicular (EF) Responses

**Figure 1:**
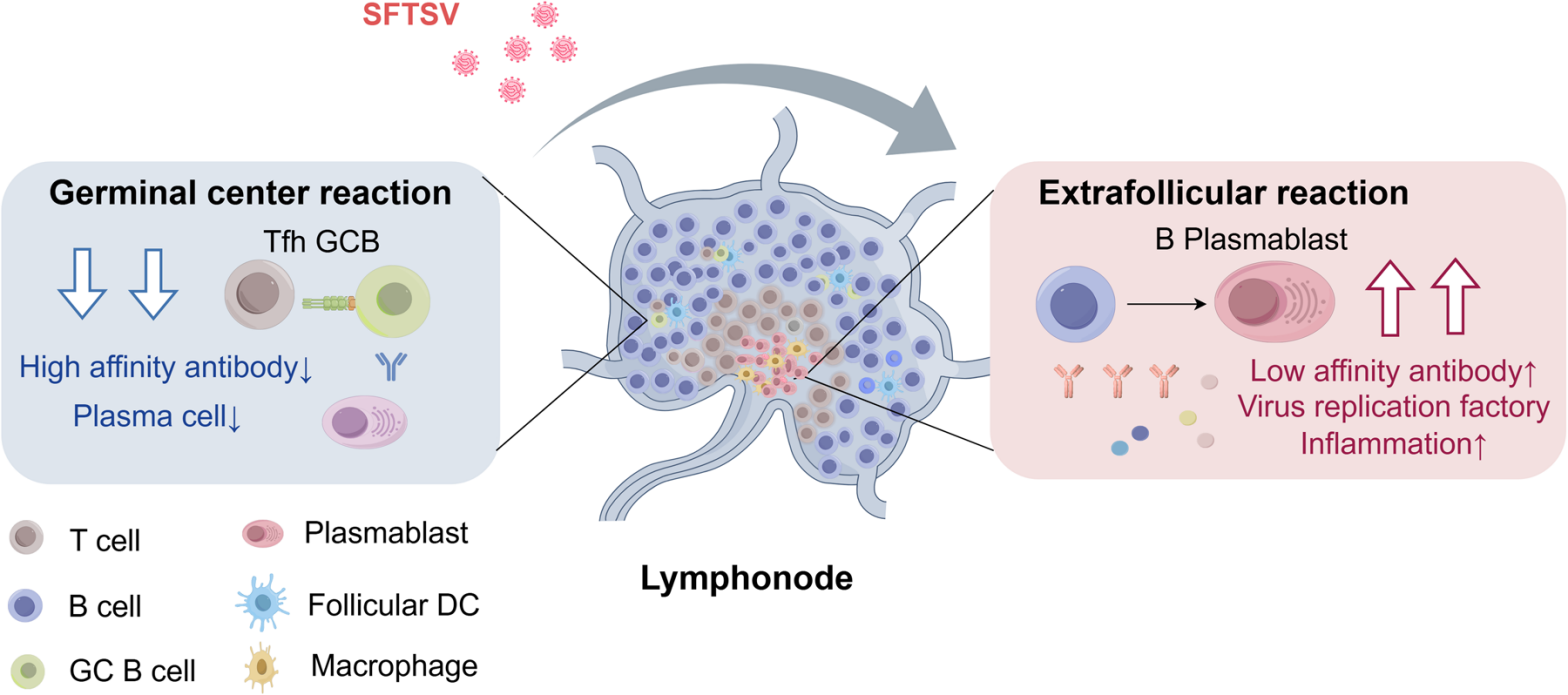
In severe SFTSV infection, excessive activation of extrafollicular (EF) responses drives robust plasmablasts proliferation, which contributes to enhanced viral replication and systemic inflammation. This dysregulated immune activation suppresses germinal center (GC) formation, ultimately impairing the production of high‐affinity, virus‐specific antibodies.

The GC and EF pathways present two distinct routes through which B cells differentiate into antibody‐secreting cells, both playing critical roles in the humoral immune response [42]. The EF pathways facilitates the rapid production of low‐affinity antibodies by plasmablasts during early infection, while the GC pathway supports the generation of high‐affinity, long‐lived plasma cells and memory B cells, thereby contributing to durable immunity. GCs are specialized microenvironment structures within secondary lymphoid organs where activated B cells undergo clonal expansion, somatic hypermutation, and affinity maturation to produce high‐affinity antibodies. SFTSV infection has been shown to disrupt this balance by promoting aberrant actibation of the EF pathway response while concurrently suppressing GC formation (Figure 1). In animal models, such as SFTSV‐infected cats, the virus induces architectural disruption of lymph nodes, characterized by decreased proliferation of Ki67^+^ cells and Bcl‐6^+^ GC B cells, alongside increased caspase‐3‐mediated apoptosis of T cells within the paracortex [43]. This GC suppression and EF dominance have also been observed in other infectious diseases, including severe acute respiratory syndrome coronavirus 2 (SARS‐CoV‐2), dengue *Salmonella* and *Ehrlichia* infections, all of which produce low‐affinity, low‐somatic mutation antibodies [44, 45, 46, 47]. Non‐neutralizing antibodies generated via EF pathways may contribute to antibody‐dependent enhancement (ADE) by promoting viral uptake through Fc receptor‐bearing cells [44]. Inflammatory cytokines such as IL‐6 and interferon‐gamma (IFN‐γ), elevated during severe infection, can impair peripheral B cell tolerance, mirmicking mechanisms observed in autoimmune diseases such as like systemic lupus erythematosus (SLE) [48]. Moreover, infections like SARS‐CoV‐2 exploit toll‐like receptor (TLR‐7) activation and neutrophil extracullular trap formation (NETosis) to drive autoantibody production and immunopathology [49]. Our previous studies in SFTS patients revealed that EF activation leads to the production of pathogenic autoantibodies derived from naive B cells, including anti‐nuclear antibodies, antiphospholipid antibodies and anti‐endothelial cell antibodies [50]. This EF‐driven autoantibody response correlates with heightened systemic inflammation, contributing to tissue damage and overall immune dysregulation in severe cases.

## 5 SFTSV Specific Antibody Production

Detection of virus‐specific antibodies play a centrol role in evaluating humoral immune responses and guiding clinical management of SFTSV infection. IgM antibodies against SFTSV typically emerge between 4 and 21 days following symptom onset (median: 9 days), peak around 4 weeks, and may persist for up to 6 months. IgG antibodies usually appear between 2 and 9 weeks post‐infection (median: 6 weeks), reach peak levels within 6 months, and can remain detectable for as long as 3 years in most patients [51]. Early IgM production is indicative of an acute‐phase response, while IgG seroconversion (commonly occurring within 14–21) correlates with recovery and the establishment of immune memory. Importantly, the absence of nucleocapsid protein (NP)‐specific IgM in the acute phase is associated with worse outcomes, including elevated viral loads, coagulation disorders, liver injury, and increased mortality [51]. Inadequente IgM or IgG responses to structural viral proteins sucha as NP and Gn are strong predictors of fatal disease, reflecting impaired neutralization and uncontrolled viral replication [17]. Paradoxically, fatal cases exhibited a marked expansion of circulating plasmablasts but fail to produce detectable levels of virus‐specific IgG, suggesting defective maturation of antibody‐secreting cells or antigen‐specific B cell depletion [17]. Autopsy studies further demonstrate that SFTSV‐infected B cells in lymph nodes are predominantly transformed B cells, with IgG‐positive subsets infiltrating multiple organs, potentially contributing to systemic immunopathology [24]. Persistent IgM beyond 45 days post‐infection is considered a negative prognostic marker, possibly reflecting immune exhaustion or ongoing viral replication. In contrast, early IgG seroconversion is associated with favorable outcomes and effecive viral clearance [52].

Cytokine networks significantly shape the persistence and quality of antibody responses during SFTSV infection. For expample, IgG titers convalescent individuals positively correlate with regulated upon activation, normal T cell expressed and secreted (RANTES), but inversely correlated with pro‐inflammatory mediators such as colony‐stimulating factor (G‐CSF) and interferon gamma‐induced protein 10 (IP‐10) [53]. The dynamics of neutralizing antibody (NAbs), measured using pseudovirus‐based assays, are strongly predictive of clinical outcomes. Among suevivors, NAbs become detectable by Day 15 post‐symptom in approximately 27% of patients (16/43), whereas neither anti‐Gn IgG or NAbs are observed in deceased patients during hospitalization. Patients with mild disease or lower viral loads tend to achieve earlier NAb seroconversion, suggesting that a prompt humoral response plays a crucial role in limiting viral dissemination. NAb titers peak around 2 months after symptom onset, decline sharply over 6 months to 4 years, and then taper off more gradually The kinetics of NAbs closely mirror that of anti‐Gn IgG, implying that anti‐Gn IgG may serve as a reliable surrogate marker for NAb activity [54]. Moreover, bispecific antibodies (BsAbs) targeting two distinct epitopes on the Gn glycoprotein have demonstrated synergistic neutralizing effects, offering improved resistance to viral immune evasion while maintaining antiviral potency [55]. These collective findings suggest that the failure to generate robust virus‐specific antibodies, particularly neutralizing IgG, in deceased patients is a critical immunological defect. However, the precise molecular mechanisms underlying B cell dysfunction and defective antibody production in SFTSV pathogenesis remain to be fully elucidated.

## 6 T Follicular Helper (Tfh) Dysregulation in SFTSV Infection

Tfh cells play a pivotal role in humoral immunity by coordinating B cell activation, GC formation, and the production of high‐affinity antibodies. This is achieved through co‐stimulatory molucules such as CD40 ligand (CD40L) and cytokine including IL‐21, IL‐4, IFN‐γ, which collectively promote B cell proliferation, class‐switch recomination and antibody affinity maturation [14]. SFTSV infection significantly impairs Tfh cell function, thereby compromising GC responses and downstream antibody production. Apoptosis of CD4^+^ T cells, along with the expansion of myeloid‐derived suppressor cells (MDSCs), contributes to a marked reduction in CXCR5^+^ Tfh cells within secondary lymphoid tissues [56, 57]. Additionally, viral infection alters GC structural integrity [43]. A decline in IL‐21 secretion from dysfunctional Tfh cells impairs B cell differentiation and antibody affinity maturation [17]. Moreover, suppression of CD40‐CD40L interactions delays IgG seroconversion and weakens neutralizing anti‐Gn antibody responses. The cytokine storm associated with SFTSV infection, marked by elevated IL‐6, IL‐10, ranulocyte colony‐stimulating factor (G‐CSF), interferon gamma‐induced protein 10 (IP‐10) and IFN‐γ, further inhibites Tfh differentiation. This inhibition likely occurs via signal transducer and activator of transcription 3 (STAT3) inhibition and NLR family pyrin domain containing 3 (NLRP3) inflammasome activation [18, 53, 58, 59]. Elevated IL‐10 and IFN‐γ levels also directly suppress B cell proliferation and interfere with CXCR4‐mediated homing of plasma cells to bone marrow niches, thereby reducing the formation of long‐lived antibody‐secreting cells [17]. Collectively, these immune disruptions severely compromise GC reaction, reduce the quality and durability of antibody responses, and hinder the establishment of immune memory. This dysregulation contributes to persistent viral replication and worsens clinical outcomes in SFTS.

## 7 Research Progress in Protein Structure Analysis for SFTSV Vaccine Design

In recent years, structural biology has significantly advanced the development of vaccines against SFTSV. High‐resolution techniques such as cryo‐electron microscopy (cryo‐EM) have enabled near‐atomic visulization of SFTSV virions, identifying the envelope glycoproteins Gc and Gn, organized as heterodimers, as major targets for neutralizing antibodies [60, 61]. Detailed structural analyses have mapped key neutralizing epitopes, particularly within its domain I of the Gn glycoprotein, providing a foundation for rational vaccine design aimed at inducing potent humoral responses [62]. Based on these insights, several vaccine platforms have been developed, including recombinant adenovirus vectors encoding Gn and/or Gc antigens [63, 64], as well as poxvirus‐based constructs, some of which are currently undergoing preclinical or clinical evaluation [65]. Preliminary studies in animal models have demonstrated that these candidates can elicit strong NAb response and attenuate viral replication [63, 64]. Looking forward, future vaccine development is expected to combine structural epitope optimization with immunomodulatory approaches, such as enhancing B‐Tfh cell interactions, to improve both breadth and durability of immuen protection across multiple SFTSV genotypes.

## 8 Future Challenges

SFTSV infection induces excessive proliferation of plasmablasts, contributing to immunopathology while failing to generate protective, high‐affinity antibody responses. This dysregulated responses highlights an urgent need for precision immunotherapeutics strategies that can selectively suppress pathogenic plasmablast activity without imparing the benifical functions of B cells essential for durable immunity. Efforts to enhance GC formation, such as using CD40 agonists or IL‐21 supplementation, have shown potential, but these approaches face limitations due to the risk of off‐target immune activation and amplication of cytokine storms. Achieving a balance between immune activation and regulation remains a critical hurdle in therapeutic design. Vaccine development also confronts major obstacles stemming from SFTSV's evolutionary adaptations. Two key mechamisms of immune evasion include: (1) Antigenic variability in surface glycoproteins Gn and Gc, particularly across different SFTSV genotypes (A‐F); (2) Glycan shielding, which masks key epitopes and reduces antibody accessibility. Althrough current neutralizing antibodies and vaccines candidates largely target conserved epitopes within the Gn domain I, structural analyses reveal significant heterogeneity in Gn/Gc antigenic landscape across strains. This variability poses challenges for inducing broad cross‐genotyope immunity. To overcome these barriers, future vaccine strategies must integrate high‐resolution structural insights to identify conserved, glycan‐unshielded epitopes, therby guiding the design of broadly protective immunogens capable of curcumventing viral immune escape.

## 9 Conclusion

Under physiological conditions, humoral immunity, coordinated by B cells, Tfh cells and cytokine networks, provided protective robust protection against pathogens through the generation of high‐affinity, antigen‐specific antibodies. However, SFTSV infection profoundly imparis this canonical humoral response, characterized by dysregulational B cell activation and excessive plasmablast expansion, which in tern excerbates immune‐mediated tissue damage and fail to confer effective viral clearance. Future research priorities should focus on elucidating the molecular mechanistic by which SFTSV subverts humoral immuity, particularly how the virus uncouples antibody quality from quantity. Advanced techniques such as scRNA‐seq and spatial transcriptomics will be instrumental in dessecting these immune evasion strategies at high resolution. Therapeutic development must strike a delicate balance between targeted immunomodulation, aimed at suppressing pathogenic B cell subsets, and vaccine strategies that leverage structural biology to target conserved, glycan‐unshielded neutralizing epitopes. Concurrently, the refinement of adjuvant systems to promote GC fidelity, rather than indiscriminate immune activation, will be essential. By addressing these key chanllenges, next‐generation immunotherapies and vaccines hold potential to shift paradigm of SFTSV management from symptom control toward disease resolutoion. Such advances may significantly improve patient outcomes and contribute to effective control and prevention of this emerging viral threat.

## Author Contributions

**Hongyan Hou:** writing – original draft. **Xu Yuan:** methodology. **Rujia Chen:** conceptualization, methodology. **Zhigang Xiong:** writing – review and editing. **Shiji Wu:** formal analysis, supervision. **Feng Wang:** writing – review and editing. All authors read and approved the final version of the manuscript.

## Conflicts of Interest

The authors declare no conflicts of interest.

## Transparency Statement

The lead author Zhigang Xiong, Shiji Wu, and Feng Wang affirms that this manuscript is an honest, accurate, and transparent account of the study being reported; that no important aspects of the study have been omitted; and that any discrepancies from the study as planned (and, if relevant, registered) have been explained.

## Data Availability

No datasets were generated or analyzed during the current study. All information discussed is based on published literature. Dr. Hongyan Hou had full access to all of the data in this study and takes complete responsibility for the integrity of the data and the accuracy of the data analysis.
